# Serum M2BPGi as a predictor of hepatocellular carcinoma development in chronic hepatitis B and C: a systematic review and meta-analysis

**DOI:** 10.3389/fmed.2026.1897953

**Published:** 2026-07-15

**Authors:** Yunchul Park, Younggoun Jo, Hyo-Sin Kim, Sola Lee, Hong-Sung Jung, Ho-Kyun Lee, Soo Jin Na Choi

**Affiliations:** Department of Surgery, Chonnam National University Medical School and Hospital, Gwangju, Republic of Korea

**Keywords:** biomarker, chronic hepatitis B, chronic hepatitis C, hepatocellular carcinoma, Mac-2 binding protein glycosylation isomer (M2BPGi), meta-analysis

## Abstract

**Background:**

Hepatocellular carcinoma (HCC) kills about 830,000 people each year, and most cases arise in chronic viral hepatitis. Better risk markers would let clinicians focus surveillance where it matters. Mac-2 binding protein glycosylation isomer (M2BPGi) is a serum glycobiomarker of liver fibrosis. Several cohort studies tie elevated M2BPGi to HCC. No quantitative synthesis has pooled this evidence across viral etiologies.

**Methods:**

We searched PubMed/MEDLINE, Embase, the Cochrane Library, Web of Science, and Scopus through April 2026 for longitudinal studies that reported hazard ratios (HRs) for HCC linked to elevated M2BPGi in chronic hepatitis B (CHB) or C (HCV). We registered the protocol with PROSPERO. The primary pool restricted to longitudinal studies with categorical cutoffs that reported HRs. We used random-effects meta-analysis with restricted maximum likelihood and the Hartung–Knapp adjustment.

**Results:**

Twenty-three publications (24 study entries) met inclusion criteria. The primary pool of 15 longitudinal categorical HR-reporting studies covered 12,161 participants and at least 1,189 HCC events. The pooled HR was 4.61 (95% CI 3.24–6.54; *p* < 0.0001) with low heterogeneity (I^2^ = 25.6%). The 95% prediction interval ran from 2.08 to 10.18. The effect held across etiology (CHB *k* = 5 HR 4.12; HCV *k* = 10 HR 5.07; p_subgroup = 0.62) and across measurement timing (baseline HR 3.21; on/post-treatment HR 5.62; post-SVR HR 5.45; p_subgroup = 0.23). Two sensitivity pools re-introducing OR-based studies gave HR 4.71 (*k* = 16) and HR 4.89 (*k* = 17). Egger’s test was positive (*p* = 0.0003); trim-and-fill imputed six studies and pulled the adjusted HR to 3.53.

**Conclusion:**

Elevated serum M2BPGi is strongly and consistently associated with HCC development in patients with chronic hepatitis B and C. The effect holds across etiology, treatment status, and measurement timing–including after sustained virological response. Funnel asymmetry suggests the true effect is closer to a 3.5-fold than a 4.6-fold risk increase, still large enough to matter clinically. M2BPGi is a credible candidate to layer onto current viral hepatitis surveillance, particularly for risk stratification after virological control.

**Systematic Review Registration:**

https://www.crd.york.ac.uk/PROSPERO/view/CRD420261372566, identifier CRD420261372566.

## Introduction

Hepatocellular carcinoma (HCC) is the sixth most common cancer and the third leading cause of cancer-related death worldwide (1, 2). Roughly 830,000 people die from it each year. Most cases arise in chronic viral hepatitis. Chronic hepatitis B (CHB) and chronic hepatitis C (HCV) together account for about half of new HCC globally. Current surveillance with ultrasound and serum alpha-fetoprotein (AFP) catches only a fraction of early-stage tumors (3–5). Better risk markers would let clinicians focus surveillance on the patients who need it most.

Mac-2 binding protein glycosylation isomer (M2BPGi) is a serum glycoprotein, measured by a Wisteria floribunda agglutinin-positive assay. It tracks liver fibrosis stage in CHB and HCV (6, 7). Over the past decade, several cohort studies have also tied elevated M2BPGi to subsequent HCC–measured at baseline, during antiviral treatment, or after sustained virological response (SVR) (8–19). The mechanism is plausible. M2BPGi reflects hepatic stellate cell activation, and progressive fibrosis is the soil from which HCC grows (20, 21).

How strong is the pooled association? We do not yet have a clear answer. The largest previous synthesis pooled 14 CHB studies on a continuous per-COI scale and reported a modest pooled HR of 1.18 per unit (22). That synthesis did not separate longitudinal categorical evidence from cross-sectional or OR-based studies, and it did not cover HCV. Since then, many new HCV cohorts and longer follow-up CHB studies have appeared. A purer, broader synthesis is overdue.

We pooled longitudinal categorical M2BPGi studies in CHB and HCV cohorts. We asked three questions. First, what is the pooled magnitude of the HCC risk associated with elevated M2BPGi? Second, does the association hold across etiology, treatment status, and measurement timing? Third, how vulnerable is the estimate to publication bias?

## Methods

### Protocol and registration

We followed the PRISMA 2020 and MOOSE checklists. We registered the protocol with PROSPERO before data extraction (CRD420261372566). The PRISMA checklist is provided in the Supplementary Material.

### Search strategy

We searched PubMed/MEDLINE, Embase, the Cochrane Library, Web of Science, and Scopus. The last search ran on April 16, 2026. We combined three groups of terms: M2BPGi (including “Mac-2 binding protein glycosylation isomer” and “WFA-positive M2BP”), HCC (“hepatocellular carcinoma” and synonyms), and chronic liver disease (including “chronic hepatitis B,” “chronic hepatitis C,” “nonalcoholic fatty liver disease,” “NAFLD,” “MASLD,” “MAFLD,” “NASH,” and “alcoholic liver disease”). We applied no language restriction. We also screened reference lists of included studies and prior reviews.

### Scope and eligibility

We screened for M2BPGi–HCC studies across all etiologies of chronic liver disease. The non-viral studies we identified did not meet the criteria for our longitudinal synthesis. In metabolic dysfunction-associated steatotic liver disease (MASLD/MAFLD/NAFLD/NASH), one retrospective study of 331 patients reported elevated M2BPGi as a predictor of HCC, but the effect was expressed as an odds ratio from multivariate logistic regression (1.57 per cut-off index unit) rather than a time-to-event hazard ratio (23); serum M2BP likewise tracks fibrosis stage rather than incident HCC in non-alcoholic fatty liver disease (24). In non-viral chronic liver disease among patients with diabetes, a cross-sectional study likewise reported higher M2BPGi in patients with HCC using an odds-ratio design (25). No non-viral study reported a longitudinal hazard ratio with a categorical M2BPGi cut-off, the effect measure that defines our primary pool, and the two measures are not interchangeable when the outcome is non-rare and follow-up varies. An etiology-spanning quantitative synthesis is therefore not yet feasible, and we restricted the present synthesis to CHB and HCV cohorts. The NAFLD study excluded during the original full-text screening, together with one further non-viral study identified in a literature search updated to June 2026 in response to peer review, are summarized in Supplementary Table 1; neither alters our conclusion that no eligible non-viral longitudinal cohort currently exists.

Studies were included if they (1) used a cohort, case-control, nested case-control, or cross-sectional design; (2) measured serum M2BPGi (WFA-positive M2BP); (3) reported HCC as an outcome with an effect estimate (HR, RR, or OR) and 95% CI, or sufficient data to compute one; and (4) enrolled adults with CHB or HCV. We excluded case reports, narrative reviews, conference abstracts without full data, and studies on nonviral etiologies.

### Study selection and data extraction

Two reviewers independently screened titles and abstracts, then full texts. Disagreements were resolved by consensus, with a third reviewer adjudicating when needed. We extracted study and population characteristics (country, design, sample size, HCC events, follow-up, treatment status, measurement timing), M2BPGi assay details (cutoff and units), and effect estimates (HR or OR with 95% CI). When studies reported both adjusted and unadjusted estimates, we used the adjusted value.

### Quality assessment

We used the Newcastle-Ottawa Scale (NOS) for observational studies. We scored selection, comparability, and outcome domains, and flagged studies with NOS ≥7 as high quality. Two reviewers scored independently.

### Statistical analysis

#### Primary effect measure

We restricted the primary pool to longitudinal studies that reported HRs with categorical M2BPGi cutoffs (*k* = 15). HRs and ORs are not interchangeable when the outcome is non-rare and follow-up varies. We therefore kept them in separate analyses. This choice prioritized methodological purity over inclusion.

#### Pooling

We used random-effects meta-analysis with the restricted maximum likelihood (REML) estimator for between-study variance (τ^2^). We applied the Hartung–Knapp adjustment to the test statistic and 95% CI, which protects against false precision in small meta-analyses. We computed the 95% prediction interval to convey the range of effects expected in a future study from a similar population.

#### Subgroup and sensitivity analyses

We pre-specified three subgroup contrasts in the primary pool: etiology (CHB vs. HCV), treatment status (treated/SVR vs. mixed), and measurement timing (baseline vs. on/post-treatment vs. post-SVR). We pre-specified two sensitivity pools that re-introduced OR-based estimates (*k* = 16: adding Liu 2017; *k* = 17: adding Vincent 2022). We ran a leave-one-out analysis on the primary pool. We pooled the seven studies reporting continuous per-1-COI effects as supplementary dose–response evidence.

#### Heterogeneity

We quantified heterogeneity with Cochran’s Q and the I^2^ statistic, using 25%, 50%, and 75% as conventional anchors for low, moderate, and high heterogeneity.

#### Publication bias

We inspected funnel plots and ran Egger’s regression for the primary pool. We applied the trim-and-fill method to estimate an adjusted effect after imputing potentially missing studies.

#### Software

All analyses ran in R version 4.3 with the meta (version 8.3) and metafor (version 4.8) packages. Two-sided *p* < 0.05 defined statistical significance.

## Results

### Literature search and study selection

**FIGURE 1:**
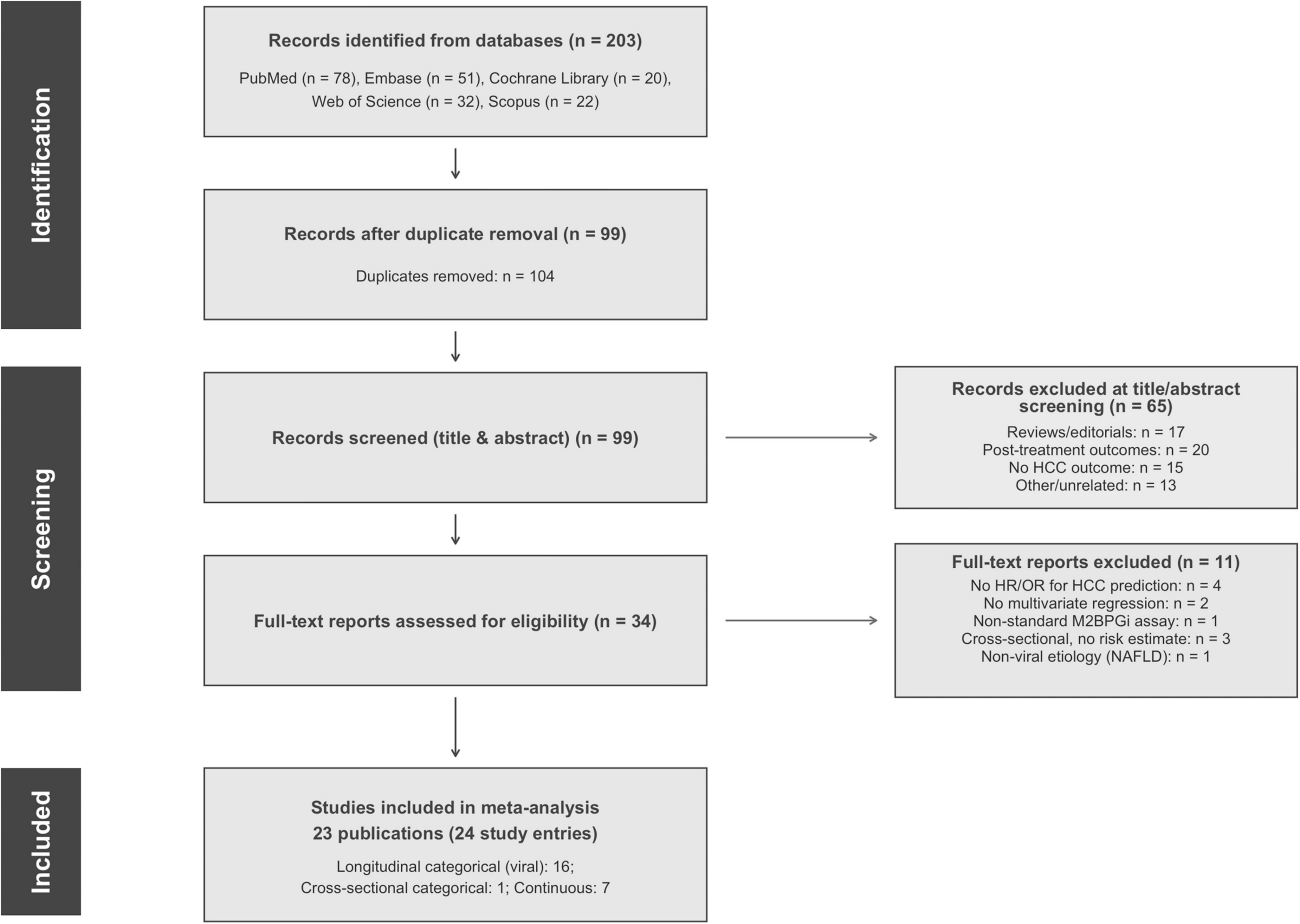
PRISMA 2020 flow diagram illustrating the literature search and study selection process. A total of 203 records were identified from five databases (PubMed, Embase, Cochrane Library, Web of Science, and Scopus). After removing 104 duplicates, 99 records were screened by title and abstract, and 34 full-text articles were assessed for eligibility. Eleven studies were excluded for the following reasons: no HR or OR reported (*n* = 4), no multivariate regression analysis (*n* = 2), non-standard M2BPGi assay (*n* = 1), cross-sectional design without a risk estimate (*n* = 3), and non-viral etiology (NAFLD, *n* = 1). A total of 23 publications (24 study entries) were included in the final meta-analysis.

The systematic search returned 203 records. After deduplication and title/abstract screening, we reviewed 34 full texts. Twenty-three publications, contributing 24 study entries, met the inclusion criteria (Figure 1). No nonviral cohort study met the criteria.

### Study characteristics

The 23 included publications enrolled 12,161 participants and recorded at least 1,189 HCC events. Studies were published between 2014 and 2026. Of the 24 entries, 14 evaluated CHB and 10 evaluated HCV. Twenty-two entries used categorical M2BPGi cutoffs; seven reported continuous (per 1 COI) effects, with five entries reporting both. Twenty entries originated in East Asia (Japan, Taiwan, Hong Kong, Korea, China), three elsewhere. Detailed study characteristics are summarized in Table 1.

**TABLE 1:** Characteristics of included studies.

Study	Country	Design	Etiology	*N*	HCC events	Treatment status	Measurement timing	Cutoff type	Cutoff (COI)	HR/OR	95% CI	NOS
Liu et al. (8)	China	NCC	CHB	1,070	357	Untreated	BL	Cat	2.0	6.46	2.58–16.18	8
Shinkai et al. (29)	Japan	RC	CHB	234	24	NA-treated	On-tx	Cat	1.22	5.07	1.69–15.19	9
Tseng et al. (11)	Taiwan	PC	CHB	899	64	NA-treated	BL	Cat	1.73	2.12	1.17–3.86	9
Murata et al. (10)	Japan	RC	CHB	147	14	NA-treated	On-tx	Cat	1.5	34.9	4.3–284.9	7
Ichikawa et al. (30)	Japan	RC	CHB	112	15	Mixed	BL	Cat	0.71	8.32	1.03–67.0	7
Chen et al. (31)	Taiwan	RC	CHB	620	115	NA-treated	On-tx	Cat	1.5	3.40	1.78–6.51	9
Vincent et al. (32)	The Gambia	CS	CHB	339	73	Untreated	BL	Cat	NA	10.1	2.6–40.2	4
Yamasaki et al. (6)	Japan	RC	HCV	707	110	Mixed	BL	Cat	4.0	8.32	1.78–38.79	9
Tamaki et al. (13)	Japan	MCC	HCV	66	14	Non-SVR	BL	Cat	4.2	4.10	1.1–15.0	7
Sasaki et al. (14)	Japan	RC	HCV	238	16	SVR (IFN)	Post-SVR	Cat	2.0	5.71	1.66–19.57	8
Sato et al. (33)	Japan	RC	HCV	355	12	SVR	Post-SVR	Cat	2.80	15.21	1.77–130.94	7
Yasui et al. (16)	Japan	RC	HCV	518	13	DAA-SVR	Post-SVR	Cat	1.75	6.00	1.8–19.4	6
Osawa et al. (15)	Japan	RC	HCV	734	24	DAA-SVR	Post-SVR	Cat	1.85	5.29	2.07–13.0	7
Takemura et al. (34)	Japan	RC	HCV	670	38	DAA-SVR	Post-tx	Cat	1.62*	7.78	2.66–22.77	8
Nakagawa et al. (35)	Japan	RC	HCV	947	26	DAA-SVR	Post-SVR	Cat	1.8	2.20	0.79–6.15	7
Sato et al. (36)	Japan	RC	HCV	522	14	SVR	Post-SVR	Cat	1.62	12.57	3.50–45.09	7
Chang et al. (17)	Taiwan	RC	HCV	704	50	DAA-SVR	BL	Cat	4.0	3.33	1.54–7.23	9
Hsu et al. (37)	Taiwan	PC	CHB	384	37	NA-treated	BL	Cont	Per COI	1.07	1.01–1.14	9
Mak et al. (9)	Hong Kong	RC	CHB	207	14	Untreated	BL	Cont	Per COI	4.67	1.30–16.80	8
Mak et al. (38)	Hong Kong	MCC	CHB	285	100	NA-treated	BL	Cont	Per COI	1.24	1.04–1.48	8
Heo et al. (15)	South Korea	RC	CHB	95	7	Mixed	BL	Cont	Per COI	2.38	1.06–5.34	7
Jun et al. (22)	South Korea	RC	CHB	714	NR	Mixed	BL	Cont	Per COI	1.11	1.05–1.18	8
Kim et al. (12)	South Korea	RC	CHB	1,323	52	Mixed	BL	Cont	Per COI	1.44	1.14–1.83	9
Su et al. (39)	Taiwan	RC	CHB	271	NR	NA-treated	On-tx	Cont	Per COI	1.58	1.19–2.10	9

NCC, nested case-control; RC, retrospective cohort; PC, prospective cohort; MCC, matched case-control; CS, cross-sectional; BL, baseline; On-tx, on-treatment; Post-tx, post-treatment; Cat, categorical; Cont, continuous; COI, cutoff index; NR, not reported; NOS, Newcastle-Ottawa Scale.

*Combined male and female estimates; HR/OR values are multivariate-adjusted unless otherwise noted.

### Quality assessment

The median NOS score was 8 (range 4–9). Twenty-two of the 24 entries (92%) were rated high quality (NOS ≥ 7). One was moderate (NOS 6) and one was low (NOS 4). Detailed NOS scores are presented in Table 2.

**TABLE 2:** Newcastle-Ottawa Scale quality assessment of included studies.

Study	S1	S2	S3	S4	C1	C2	O1	O2	O3	Total	Quality
Liu et al. (8)	1	1	1	1	1	1	1	0	1	8	High
Shinkai et al. (29)	1	1	1	1	1	1	1	1	1	9	High
Tseng et al. (11)	1	1	1	1	1	1	1	1	1	9	High
Murata et al. (10)	1	1	1	1	1	0	1	1	0	7	High
Ichikawa et al. (30)	1	1	1	1	1	0	1	1	0	7	High
Chen et al. (31)	1	1	1	1	1	1	1	1	1	9	High
Vincent et al. (32)	0	1	1	0	1	0	1	0	0	4	Low
Yamasaki et al. (6)	1	1	1	1	1	1	1	1	1	9	High
Tamaki et al. (13)	1	1	1	1	1	1	1	0	0	7	High
Sasaki et al. (14)	1	1	1	1	1	0	1	1	1	8	High
Sato et al. (33)	1	1	1	1	1	0	1	1	0	7	High
Yasui et al. (16)	1	1	1	1	1	0	1	0	0	6	Moderate
Osawa et al. (15)	1	1	1	1	1	1	1	0	0	7	High
Takemura et al. (34)	1	1	1	1	1	1	1	1	0	8	High
Nakagawa et al. (35)	1	1	1	1	1	0	1	1	0	7	High
Sato et al. (36)	1	1	1	1	1	0	1	1	0	7	High
Chang et al. (17)	1	1	1	1	1	1	1	1	1	9	High
Hsu et al. (37)	1	1	1	1	1	1	1	1	1	9	High
Mak et al. (9)	1	1	1	1	1	0	1	1	1	8	High
Mak et al. (38)	1	1	1	1	1	1	1	0	1	8	High
Heo et al. (15)	1	1	1	1	1	0	1	1	0	7	High
Jun et al. (22)	1	1	1	1	1	1	1	1	0	8	High
Kim et al. (12)	1	1	1	1	1	1	1	1	1	9	High
Su et al. (39)	1	1	1	1	1	1	1	1	1	9	High

S1, representativeness of exposed cohort; S2, selection of non-exposed; S3, ascertainment of exposure; S4, outcome not present at start; C1–C2, comparability (1 = adjusted for age/sex, 2 = additional adjustment); O1, outcome assessment; O2, adequate follow-up length; O3, adequacy of follow-up. Total score range 0–9; High quality ≥ 7, Moderate 5–6, Low ≤ 4.

### Primary meta-analysis

**FIGURE 2:**
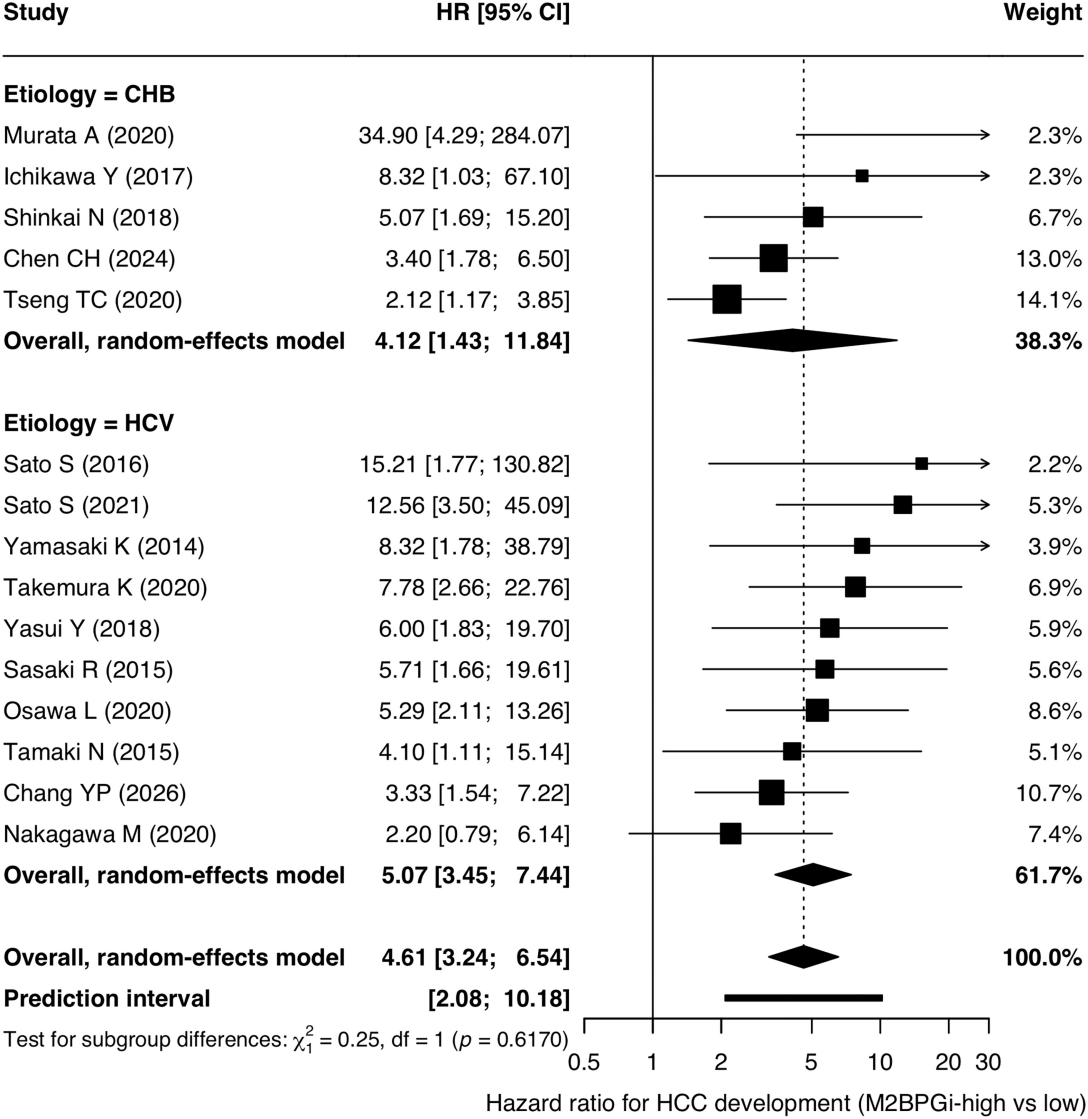
Forest plot of the 15 HR-reporting longitudinal categorical M2BPGi studies (primary analysis) stratified by etiology (CHB vs. HCV). The pooled HR was 4.12 (95% CI: 1.43–11.84) for CHB and 5.07 (95% CI: 3.45–7.44) for HCV (p for subgroup difference = 0.62). Overall pooled HR = 4.61 (95% CI: 3.24–6.54).

The primary pool included 15 longitudinal categorical HR-reporting studies. The pooled HR for HCC associated with elevated M2BPGi was 4.61 (95% CI 3.24–6.54; *p* < 0.0001) (Figure 2). Heterogeneity was low (I^2^ = 25.6%; *Q* = 18.81, *p* = 0.17). The 95% prediction interval ran from 2.08 to 10.18, supporting a clinically meaningful effect even at the lower bound. Detailed pooled estimates across all analyses are presented in Table 3. A bias-corrected estimate accounting for funnel asymmetry is reported under Publication Bias.

**TABLE 3:** Summary of meta-analysis results by analysis type and subgroup.

Analysis	k	Pooled HR	95% CI	I^2^/*p*
Primary analysis (longitudinal categorical, HR-reporting)				
Pooled HR for HCC	15	4.61	3.24–6.54	I^2^ = 25.6%; *p* < 0.0001
Subgroup by etiology (primary k = 15)				
Chronic hepatitis B (CHB)	5	4.12	1.43–11.84	I^2^ = 51.9%
Chronic hepatitis C (HCV)	10	5.07	3.45–7.44	I^2^ = 0.0%
P for subgroup difference	–	–	–	0.62
Subgroup by treatment status (primary k = 15)				
Treated/SVR	13	4.42	3.01–6.50	–
Mixed*	2	8.32	8.31–8.33	–
P for subgroup difference	–	–	–	0.0004*
Subgroup by measurement timing (primary k = 15)				
Baseline	5	3.21	1.69–6.11	I^2^ = 4.6%
On/post-treatment	4	5.62	1.70–18.62	I^2^ = 51.3%
Post-SVR	6	5.45	2.79–10.67	I^2^ = 0.0%
P for subgroup difference	–	–	–	0.23
Sensitivity analyses				
Add Liu (8) (OR-based longitudinal)	16	4.71	3.40–6.53	I^2^ = 23.6%
Add Vincent (32) (OR-based cross-sectional)	17	4.89	3.55–6.73	I^2^ = 24.1%
Continuous (per 1 COI increase)	7	1.28	1.01–1.62	I^2^ = 72.3%
HR-only continuous	5	1.28	0.96–1.70	–
Descriptive overall (all 24 entries)	24	3.33	2.33–4.77	I^2^ = 85.3%
Leave-one-out range (primary)	15	4.24–4.92	–	–
Publication bias adjustment				
Egger’s regression (primary)	15	–	–	*p* = 0.0003
Trim-and-fill adjusted (6 imputed)	21	3.53	2.53–4.93	–

*The mixed-population subgroup-difference *p*-value (0.0004) is a mechanical artifact of two studies with near-identical point estimates and τ^2^ = 0; it is reported for completeness and should be interpreted descriptively. The untreated subgroup is absent from the primary pool because the only untreated longitudinal study [Liu et al. (8)] was OR-based and was therefore reserved for the *k* = 16 sensitivity analysis. CHB, chronic hepatitis B; CI, confidence interval; COI, cutoff index; HCC, hepatocellular carcinoma; HCV, chronic hepatitis C; HR, hazard ratio; OR, odds ratio; SVR, sustained virological response.

### Subgroup analysis by etiology

The association held in both etiologies. The pooled HR in CHB (*k* = 5) was 4.12 (95% CI 1.43–11.84), and in HCV (*k* = 10) it was 5.07 (95% CI 3.45–7.44) (Figure 2). The test for subgroup difference was not significant (p_subgroup = 0.62). CHB studies showed moderate within-subgroup heterogeneity (I^2^ = 51.9%); HCV studies showed none (I^2^ = 0%).

### Subgroup analysis by treatment status

**FIGURE 3:**
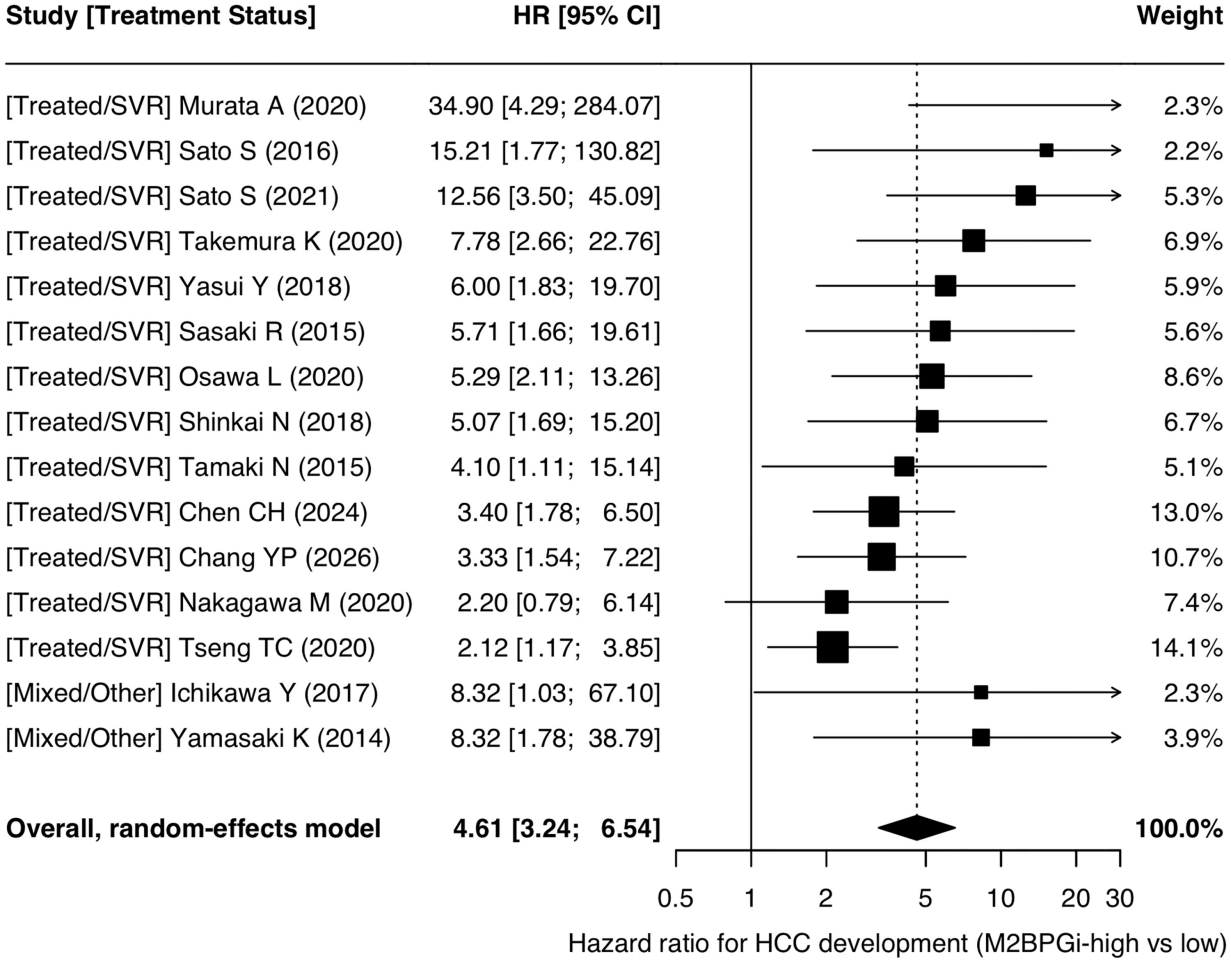
Forest plot of the 15 HR-reporting longitudinal categorical studies (primary analysis) stratified by treatment status (treated/SVR vs. mixed). The untreated subgroup is absent in the primary set because the only longitudinal untreated study (Liu 2017) was OR-based and therefore reserved for the categorical longitudinal sensitivity analysis. The Mixed subgroup contained only two studies with nearly identical point estimates and should be interpreted descriptively.

Treated/SVR studies (*k* = 13) yielded a pooled HR of 4.42 (95% CI 3.01–6.50). The mixed-population subgroup (*k* = 2) yielded a pooled HR of 8.32 (95% CI 8.31–8.33) (Figure 3). The mixed subgroup difference was statistically significant (p_subgroup = 0.0004), but this is a mechanical artifact of two studies with near-identical point estimates and τ^2^ = 0. We treat it as descriptive only. No untreated longitudinal HR-only cohort entered the primary pool; the only untreated longitudinal study (Liu 2017) was OR-based and entered the *k* = 16 sensitivity pool.

### Subgroup analysis by measurement timing

**FIGURE 4:**
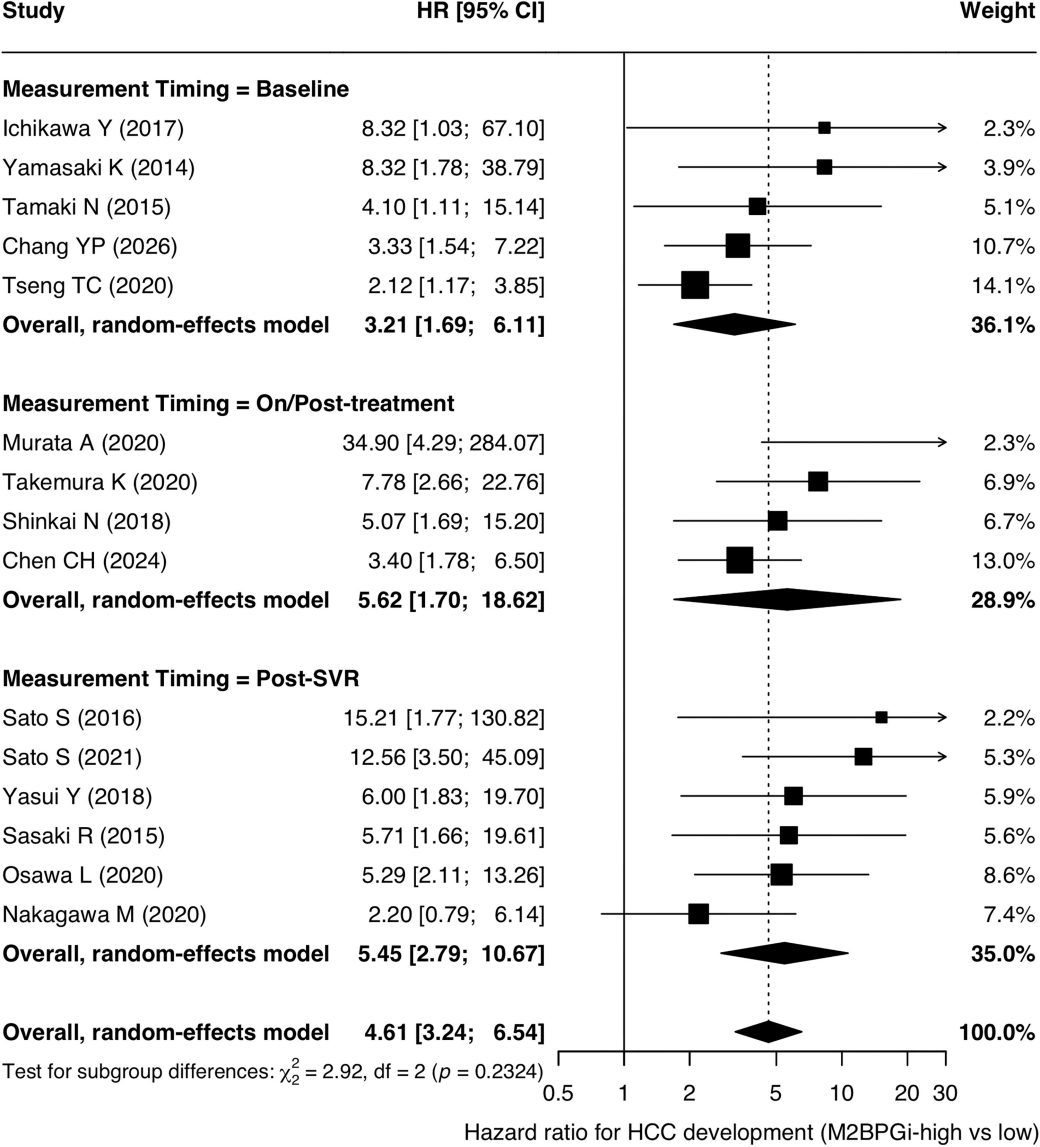
Forest plot of the 15 HR-reporting longitudinal categorical studies (primary analysis) stratified by measurement timing (baseline vs. on/post-treatment vs. post-SVR). The test for subgroup differences was not significant (*p* = 0.23).

The association held across measurement timepoints: baseline (*k* = 5) HR 3.21 (95% CI 1.69–6.11; I^2^ = 4.6%); on/post-treatment (*k* = 4) HR 5.62 (95% CI 1.70–18.62; I^2^ = 51.3%); post-SVR (*k* = 6) HR 5.45 (95% CI 2.79–10.67; I^2^ = 0%) (Figure 4). The test for subgroup difference was not significant (p_subgroup = 0.23).

### Sensitivity analyses

Leave-one-out analysis on the primary pool moved the pooled HR within a narrow range (4.24–4.92), with no single study driving the result (Supplementary Figure 5).

Adding the OR-based longitudinal CHB study Liu 2017 (*k* = 16) yielded HR 4.71 (95% CI 3.40–6.53; I^2^ = 23.6%). Adding the OR-based cross-sectional HCV study Vincent 2022 (*k* = 17) yielded HR 4.89 (95% CI 3.55–6.73; I^2^ = 24.1%). Both sensitivity pools moved the estimate marginally upward and left heterogeneity low.

Seven studies reporting continuous per-1-COI effects gave a pooled HR of 1.28 (95% CI 1.01–1.62; I^2^ = 72.3%) (Supplementary Figure 2). When restricted to HR-only continuous studies (*k* = 5), the estimate was attenuated to 1.28 (95% CI 0.96–1.70), losing statistical significance. A descriptive pool of all 24 entries yielded HR 3.33 (95% CI 2.33–4.77; I^2^ = 85.3%); this pool mixes effect measures and is reported for completeness only (Supplementary Figures 1, 4).

### Effect modification by cut-off and cirrhosis prevalence

The study-specific M2BPGi cut-off varied widely across the primary pool (0.71–4.2 COI; median 1.75 COI). In random-effects meta-regression, the cut-off did not modify the pooled hazard ratio (per 1-COI increase, ratio of hazard ratios 0.96, 95% CI 0.66–1.39; *p* = 0.80) and explained none of the between-study heterogeneity (R^2^ = 0%). A median split gave concordant pooled estimates for lower-cut-off (*k* = 7; HR 5.41, 95% CI 2.50–11.72) and higher-cut-off studies (*k* = 8; HR 4.39, 95% CI 2.96–6.51; subgroup difference *p* = 0.56). The association between elevated M2BPGi and HCC was therefore robust to the choice of cut-off (Supplementary Figure 6 and Supplementary Table 2).

Cohort cirrhosis prevalence, reported in 8 of the 15 primary studies (range 8.8%–100%), likewise did not modify the pooled hazard ratio (per 10% increase in prevalence, ratio of hazard ratios 0.97, 95% CI 0.89–1.05; *p* = 0.36; R^2^ = 0%) (Supplementary Figure 7 and Supplementary Table 2). Because no included study reported hazard ratios stratified by individual cirrhosis status, a formal cirrhotic-versus-non-cirrhotic subgroup meta-analysis was not feasible; this cohort-level meta-regression is the closest available approximation and should be interpreted as exploratory.

### Publication bias

Egger’s regression for the 15 primary studies was significant (*p* = 0.0003), indicating funnel asymmetry (Supplementary Figure 3). Trim-and-fill imputed six left-side studies, and the adjusted pooled HR fell to 3.53 (95% CI 2.53–4.93). We regard 4.61 as the observed pooled estimate and 3.53 as a conservative, bias-corrected lower bound; the true association most plausibly lies between these values, that is, a 3.5- to 4.6-fold increase in HCC risk.

## Discussion

We asked three questions about serum M2BPGi and HCC risk in chronic viral hepatitis, and we answered them in turn. First, how strong is the pooled association? Patients with elevated M2BPGi developed HCC at about four to five times the rate of patients with low M2BPGi (HR 4.61, 95% CI 3.24–6.54). Second, does the association hold across important clinical strata? It does. The pooled effect was preserved across etiology (CHB and HCV), treatment status (treated/SVR), and measurement timing (baseline through post-SVR). Third, how vulnerable is the estimate to publication bias? Egger’s test was positive and trim-and-fill pulled the adjusted HR to 3.53. The corrected estimate is still large enough to matter clinically.

The first methodological message comes from holding the primary pool to HR-reporting longitudinal categorical studies (*k* = 15) and resisting the temptation to mix in OR-based or cross-sectional evidence. The earlier synthesis by Witarto et al. pooled a continuous per-COI effect of 1.18 (95% CI 1.05–1.32) per unit, restricted to CHB (22). That choice answered a different question. By drawing a hard line at adjusted HRs with categorical cutoffs, we kept the primary estimate interpretable as “risk above versus below a clinical cutoff” rather than “risk per arbitrary unit of an assay.” Heterogeneity dropped to I^2^ = 25.6%–a striking value for a meta-analysis spanning 12 years, two etiologies, and four continents. When we relaxed the criterion in sensitivity analyses (*k* = 16, *k* = 17), the estimate barely moved (HR 4.71 and 4.89). Purity here cost almost nothing and bought interpretability.

The second message is that the effect is biologically and clinically generalizable within the constraints of the evidence base. M2BPGi reflects hepatic stellate cell activation and fibrogenesis (6, 7). The same fibrogenic mechanism drives HCC in both CHB and HCV (20, 21). The pooled effect in CHB (HR 4.12) and HCV (HR 5.07) was statistically indistinguishable (p_subgroup = 0.62). The post-SVR subgroup is the most clinically interesting: even after virological control, patients with elevated M2BPGi developed HCC at five times the rate of patients with low M2BPGi (HR 5.45, 95% CI 2.79–10.67). The risk does not reset when the virus is suppressed. This matches the broader literature showing residual HCC risk after HCV eradication in patients with advanced fibrosis (26, 27). Although the point estimate was numerically higher in HCV than in CHB, this difference was not statistically significant (p_subgroup = 0.62) and should not be over-interpreted; it may reflect the higher M2BPGi cut-offs adopted in the HCV baseline studies (around 4.0 COI versus 1.2–2.0 COI in most CHB studies) and differences in fibrogenic and glycosylation dynamics between the two infections rather than a genuine etiology effect.

A key question raised in review is whether M2BPGi merely restates liver fibrosis stage, which is itself the strongest determinant of HCC risk; if so, it would add little to existing fibrosis markers such as FIB-4 or liver-stiffness elastography. Several features of our data argue for incremental value. The primary-pool effects were adjusted, multivariable hazard ratios rather than crude associations, although the covariate sets were not uniform across studies. M2BPGi remained strongly predictive after sustained virological response (HR 5.45), a setting in which hepatic fibrosis frequently regresses–so the signal does not track static fibrosis burden in lockstep. Neither the study-specific cut-off nor cohort cirrhosis prevalence modified the pooled effect, as would be expected if M2BPGi carried information beyond the degree of fibrosis alone. Mechanistically, M2BPGi is more than a structural fibrosis gauge: its *Wisteria floribunda* agglutinin-reactive glycosylation is produced by activated hepatic stellate cells and reflects a fibro-inflammatory, pro-tumorigenic microenvironment rather than collagen quantity *per se* (6, 7, 28). Together these observations suggest M2BPGi adds prognostic information to purely structural measures such as FIB-4 and elastography. We caution, however, that the non-uniform adjustment sets preclude isolating its effect conditional on a single fibrosis covariate, and that rigorous head-to-head and combined-model comparisons (M2BPGi with FIB-4, elastography, and GALAD) remain scarce; individual-participant-data studies are needed to settle the incremental-value question.

Mac-2 binding protein glycosylation isomer is a credible addition to current HCC surveillance in chronic viral hepatitis. Three features make it attractive. It is a single blood test. It tracks the same fibrogenic pathway that ultrasound and elastography monitor. And it carries prognostic signal that is independent of viral status. We see the immediate use case as risk stratification within surveillance: identify post-SVR or treatment-stable patients whose M2BPGi is high, and direct them to more intensive imaging schedules. Patients with consistently low M2BPGi may be candidates for de-intensified follow-up. Even after the trim-and-fill correction, a 3.5-fold risk increase justifies stratification.

We see four limitations. First, nearly all included studies (23 of 24) were from East Asia, where the M2BPGi (HISCL) assay was developed and is reimbursed. The biology of fibrogenesis is conserved, but as reference ranges and optimal thresholds for serum fibrosis markers such as FIB-4 and the ELF test differ between Western and East Asian populations, the M2BPGi cut-offs calibrated in these Asian viral-hepatitis cohorts (0.71–4.2 COI) may not transfer directly to other populations; external validation outside East Asia is a priority. Second, we restricted the synthesis to CHB and HCV. We screened for M2BPGi–HCC studies in MASLD, NAFLD, NASH, and ALD; the non-viral studies we found reported associations as odds ratios from cross-sectional or logistic-regression designs (23, 25) rather than longitudinal hazard ratios, and none met our primary inclusion criteria (Supplementary Table 1). M2BPGi originated as a fibrosis biomarker in viral hepatitis (7, 28), and longitudinal cohorts coupling M2BPGi to incident HCC have so far accumulated almost exclusively in CHB and HCV; comparable time-to-event data in non-viral disease are only beginning to emerge. This gap reflects the current evidence base rather than a flaw of the present study, but it means our estimates should not be extrapolated to MASLD or ALD without dedicated cohorts. Third, funnel asymmetry was significant. Small-study effects, selective reporting, or genuine clinical heterogeneity in cutoffs likely contributed. Fourth, the mixed-population subgroup result and the descriptive overall pool both mix effect measures or sample sizes and should be read as exploratory. Future work should validate M2BPGi outside East Asia, define cohort-specific cutoffs, accumulate longitudinal MASLD and ALD evidence so an etiology-spanning meta-analysis becomes feasible, and test M2BPGi against and alongside FIB-4, the ELF test, and the GALAD score in head-to-head designs.

## Conclusion

Elevated serum M2BPGi is associated with an approximately fourfold increase in HCC risk in patients with chronic hepatitis B or C. The association is consistent across etiology, treatment status, and measurement timing, including after sustained virological response. Funnel asymmetry suggests the corrected effect is closer to a 3.5-fold increase–still large enough to be clinically actionable. M2BPGi is a credible candidate to layer onto current viral hepatitis surveillance, particularly for risk stratification after virological control. Validation outside East Asia and the accumulation of nonviral cohort evidence are the next steps.

## Data Availability

The original contributions presented in this study are included in this article/Supplementary material, further inquiries can be directed to the corresponding authors.

## Supplementary material

Supplementary Figure 1 Exploratory pool of all 24 study entries (categorical + continuous overall pool) stratified by M2BPGi cutoff type (categorical vs. continuous). The pooled HR for categorical entries was 4.89 (95% CI: 3.55–6.73; *k* = 17, all categorical sensitivity) and for continuous entries was 1.28 (95% CI: 1.01–1.62; *k* = 7). The combined pool is reported descriptively only because of high heterogeneity arising from combining different effect-size scales.

Supplementary Figure 2 Forest plot of 7 continuous M2BPGi studies (per 1 COI increase), all from CHB populations (no HCV continuous studies were available). Overall pooled HR was 1.28 (95% CI: 1.01–1.62). When restricted to HR-only continuous studies (*k* = 5), this estimate was attenuated to 1.28 (95% CI: 0.96–1.70), losing statistical significance.

Supplementary Figure 3 Funnel plot and trim-and-fill analysis for the 15 primary studies. Egger’s test indicated significant asymmetry (*p* = 0.0003). Six potentially missing studies were imputed on the left side of the funnel, yielding an adjusted HR of 3.53 (95% CI: 2.53–4.93).

Supplementary Figure 4 Funnel plot of all 24 study entries (exploratory).

Supplementary Figure 5 Leave-one-out sensitivity analysis for the 15 primary studies. The pooled HR ranged from 4.24 to 4.92 upon sequential exclusion of each study.

Supplementary Figure 6 Random-effects meta-regression of the pooled hazard ratio on the study-specific M2BPGi cut-off for the 15 primary studies. Each bubble represents one study and is sized by its meta-analytic weight; the solid line is the fitted regression and the dashed lines its 95% confidence band. The cut-off did not modify the pooled effect (per 1-COI increase, ratio of hazard ratios 0.96, 95% CI 0.66–1.39; *p* = 0.80) and explained none of the between-study heterogeneity (R^2^ = 0).

Supplementary Figure 7 Random-effects meta-regression of the pooled hazard ratio on cohort cirrhosis prevalence for the 8 primary studies that reported it (prevalence range 8.8%–100%). Each bubble represents one study and is sized by its meta-analytic weight; the solid line is the fitted regression and the dashed lines its 95% confidence band. Cirrhosis prevalence did not modify the pooled effect (per 10% increase in prevalence, ratio of hazard ratios 0.97, 95% CI 0.66–1.39; R^2^ = 0).
