# Supplementary material for: Serum M2BPGi as a predictor of hepatocellular carcinoma development in chronic hepatitis B and C: a systematic review and meta-analysis
Source: Front Med (Lausanne). 2026 Jul 15;13:1897953. doi: 10.3389/fmed.2026.1897953 (PMC13416451; doi:10.3389/fmed.2026.1897953)
Supplement: Supplementary file 1 [file Data_Sheet_1.pdf]

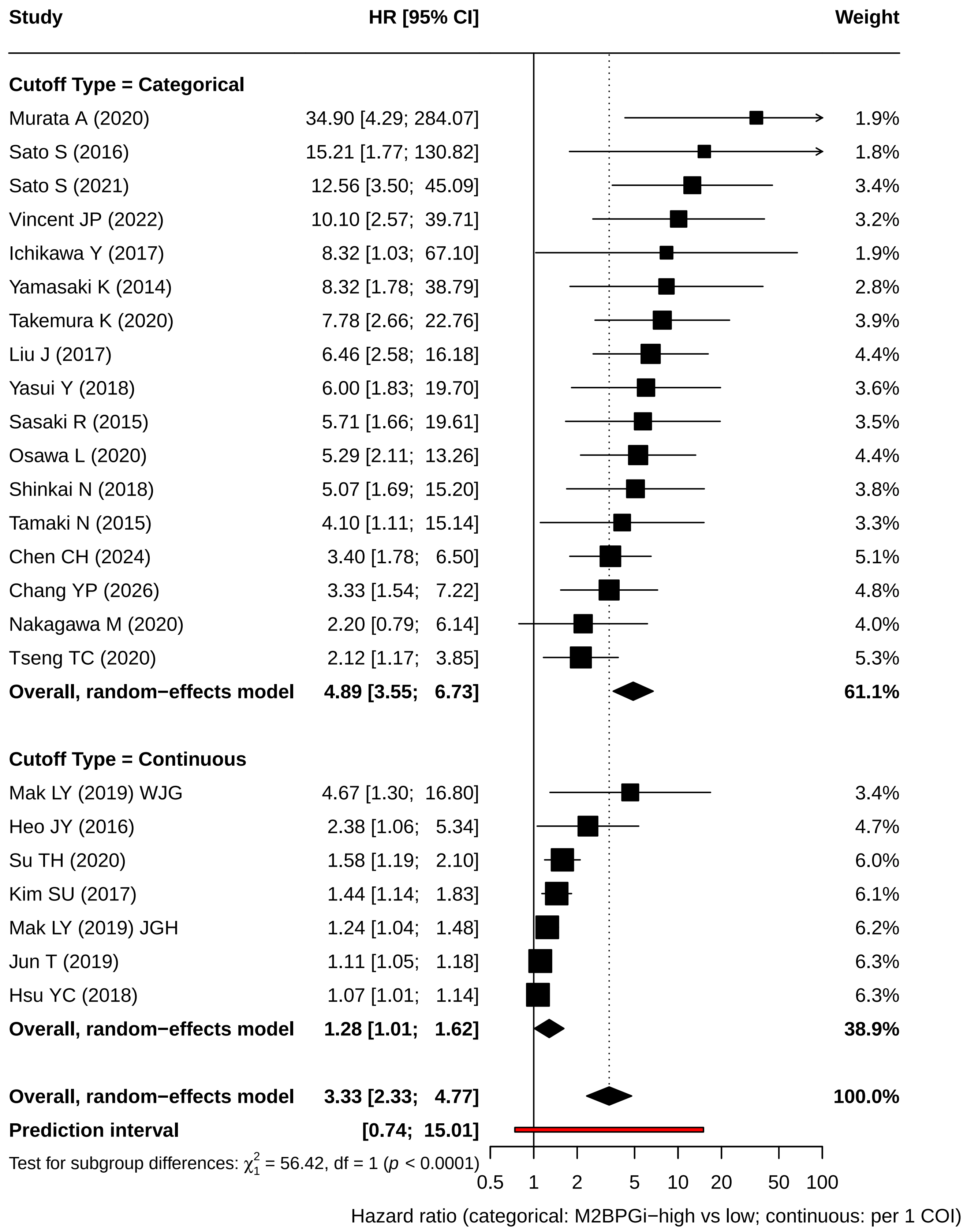

Random−effects model. Heterogeneity: I^2 = 85.3%, τ^2 = 0.4980, p < 0.0001.

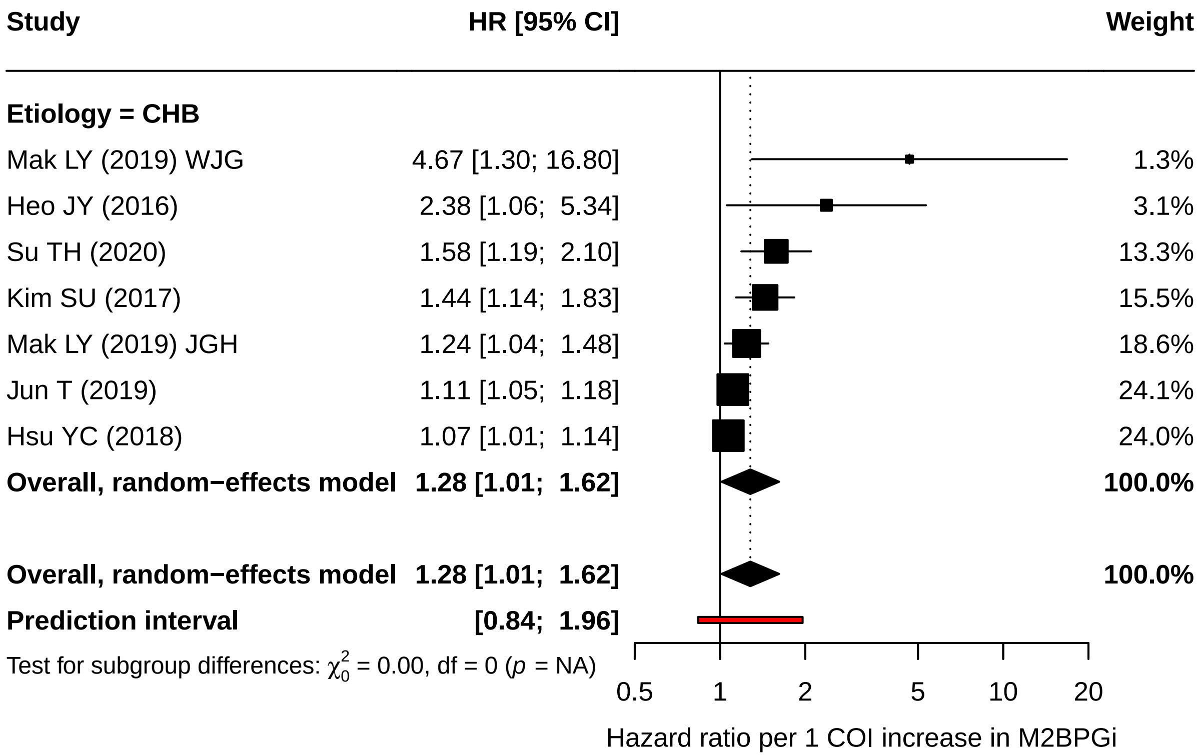

Random−effects model. Heterogeneity: I^2 = 72.3%, τ^2 = 0.0241, p = 0.0014.

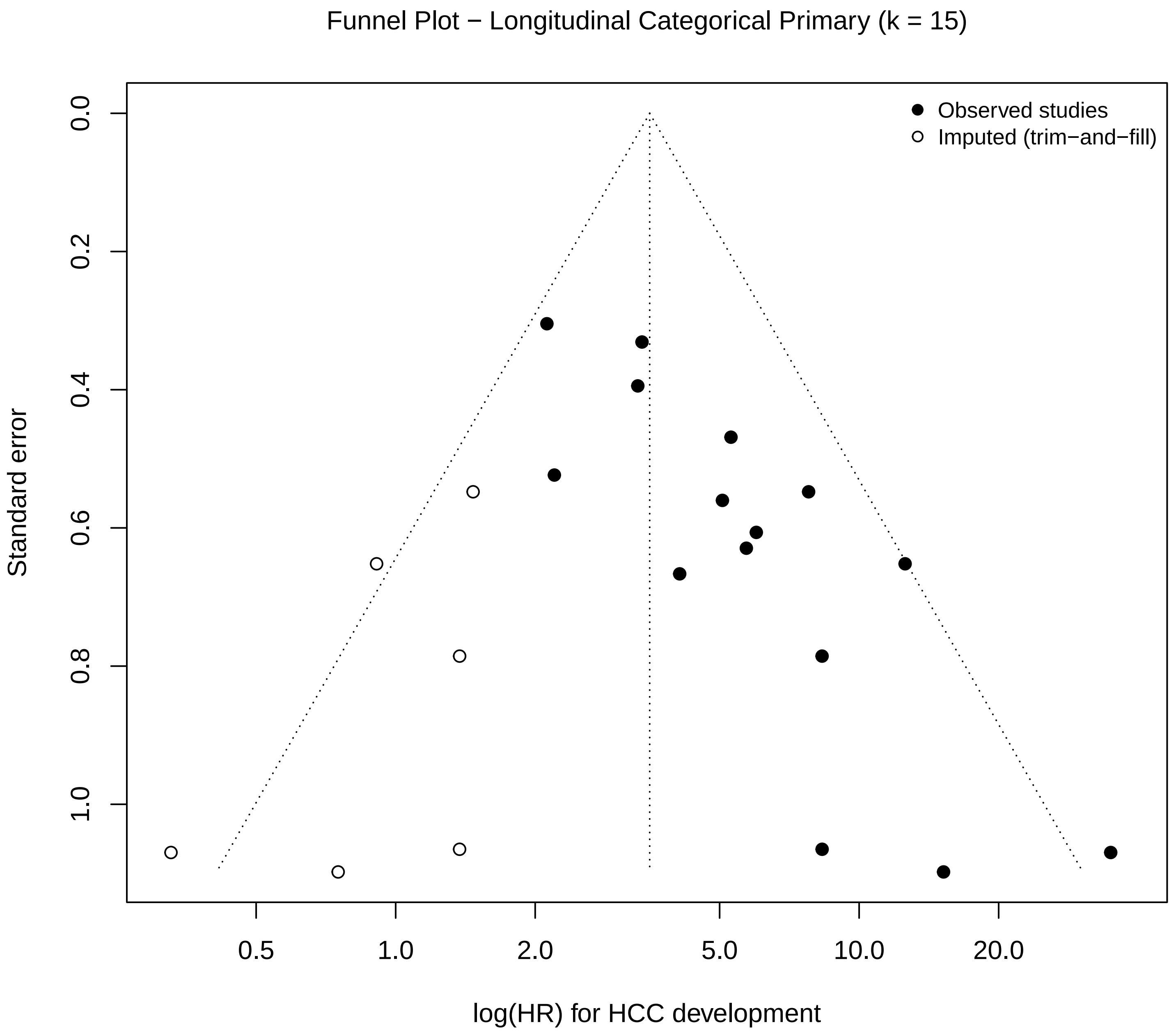

Funnel Plot − Longitudinal Categorical Primary (k = 15)
Observed studies
Imputed (trim−and−fill)
Standard error
0.0
0.2
0.4
0.6
0.8
1.0
0.5
1.0
2.0
5.0
10.0
20.0
log(HR) for HCC development

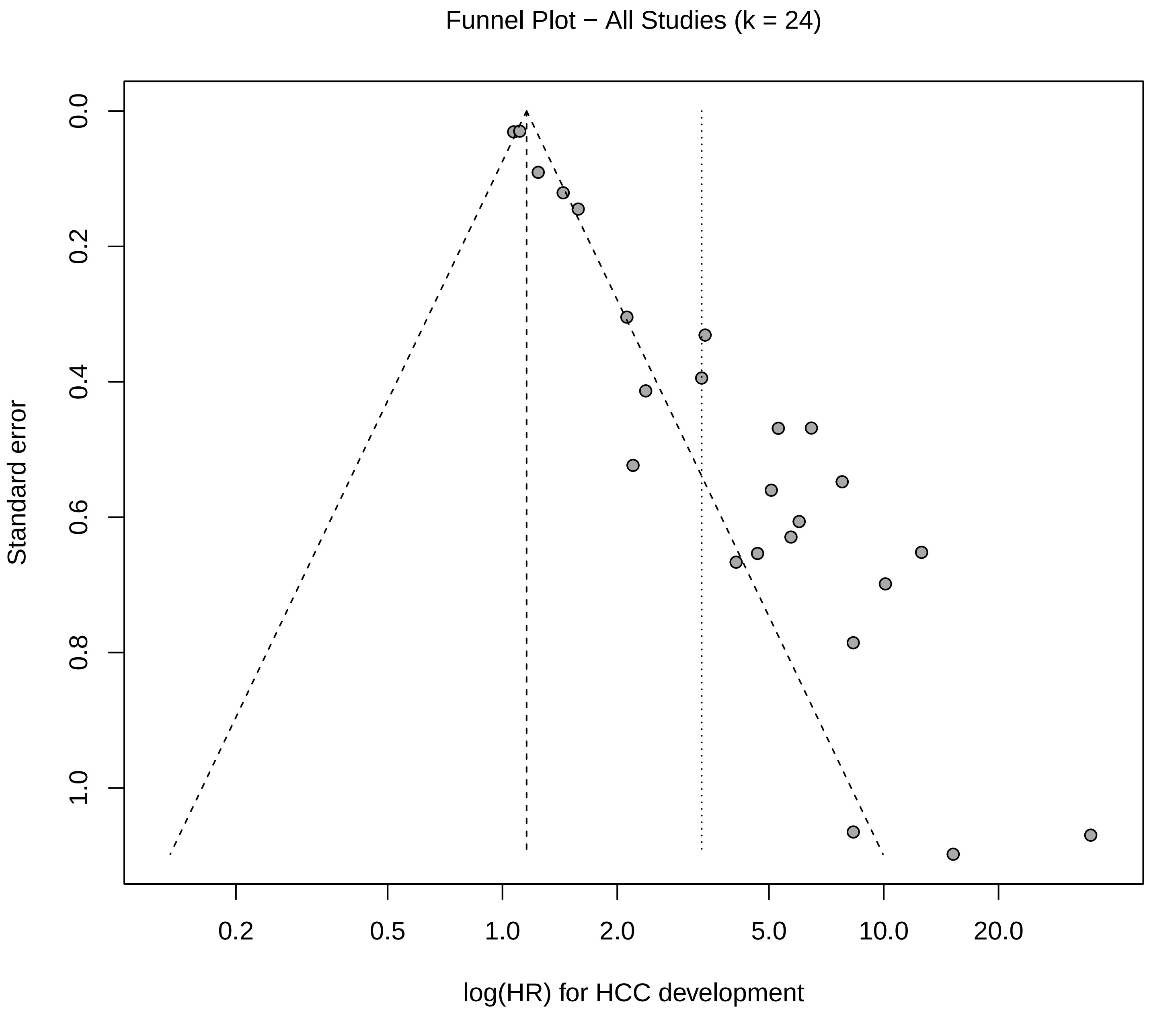
Funnel Plot − All Studies (k = 24)
Standard error
0.0
0.2
0.4
0.6
0.8
1.0
0.2
0.5
1.0
2.0
5.0
10.0
20.0
log(HR) for HCC development

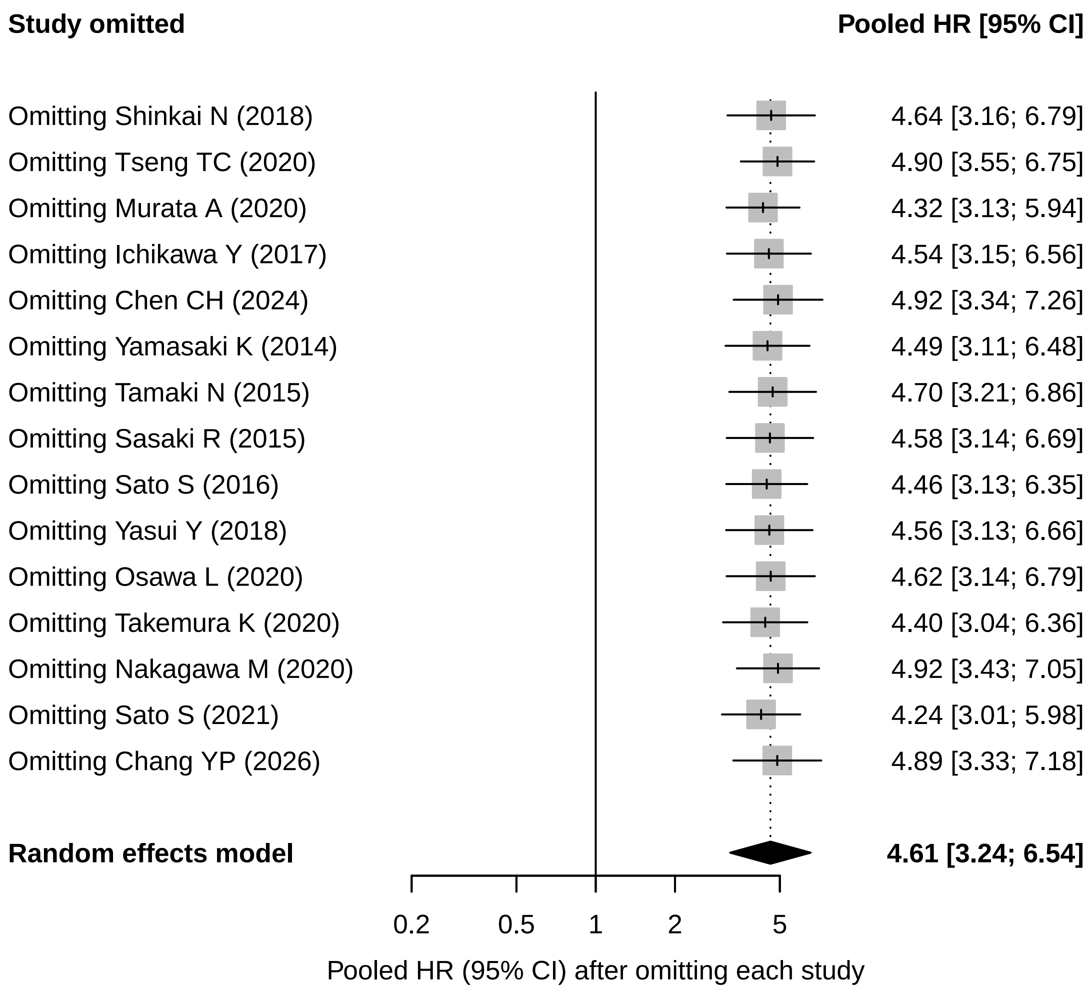

Study omitted
Pooled HR [95% CI]
Omitting Shinkai N (2018) 4.64 [3.16; 6.79]
Omitting Tseng TC (2020) 4.90 [3.55; 6.75]
Omitting Murata A (2020) 4.32 [3.13; 5.94]
Omitting Ichikawa Y (2017) 4.54 [3.15; 6.56]
Omitting Chen CH (2024) 4.92 [3.34; 7.26]
Omitting Yamasaki K (2014) 4.49 [3.11; 6.48]
Omitting Tamaki N (2015) 4.70 [3.21; 6.86]
Omitting Sasaki R (2015) 4.58 [3.14; 6.69]
Omitting Sato S (2016) 4.46 [3.13; 6.35]
Omitting Yasui Y (2018) 4.56 [3.13; 6.66]
Omitting Osawa L (2020) 4.62 [3.14; 6.79]
Omitting Takemura K (2020) 4.40 [3.04; 6.36]
Omitting Nakagawa M (2020) 4.92 [3.43; 7.05]
Omitting Sato S (2021) 4.24 [3.01; 5.98]
Omitting Chang YP (2026) 4.89 [3.33; 7.18]
Random effects model 4.61 [3.24; 6.54]
0.2
0.5
1
2
5
Pooled HR (95% CI) after omitting each study

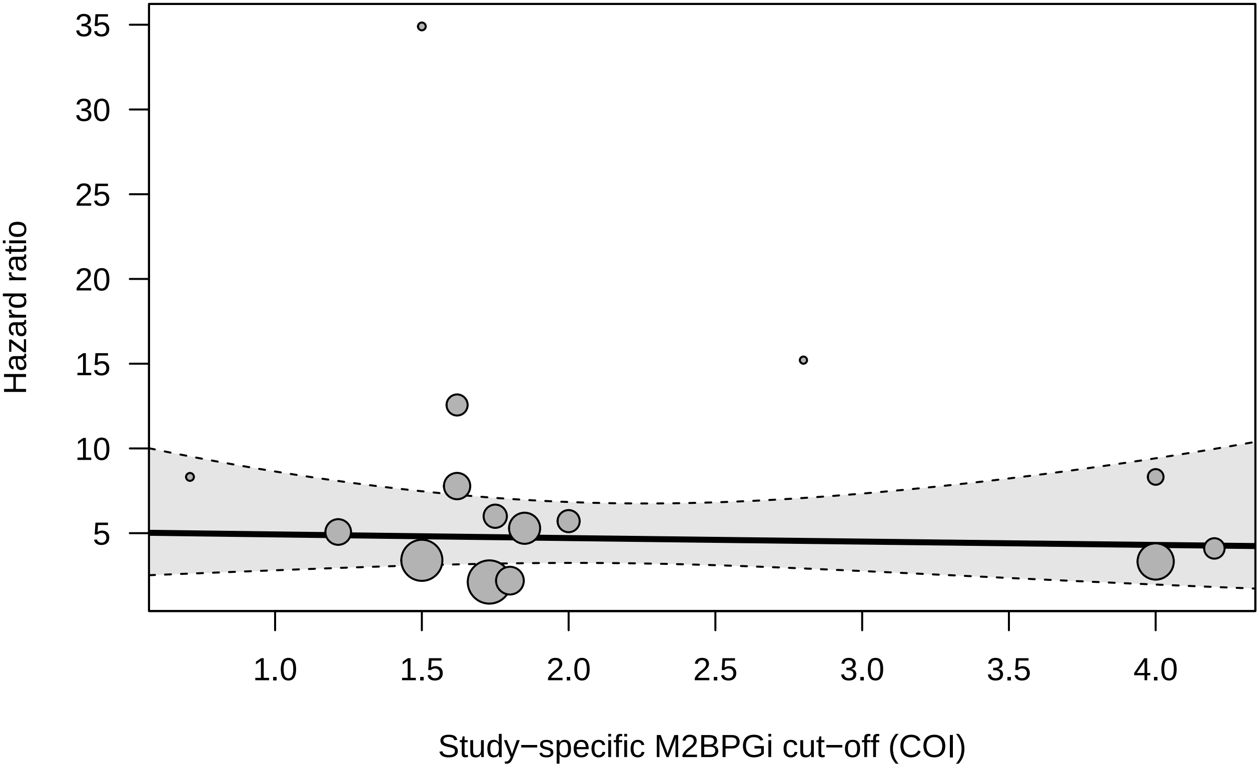

35
30
25
20
15
10
5
Hazard ratio
1.0
1.5
2.0
2.5
3.0
3.5
4.0
Study−specific M2BPGi cut−off (COI)

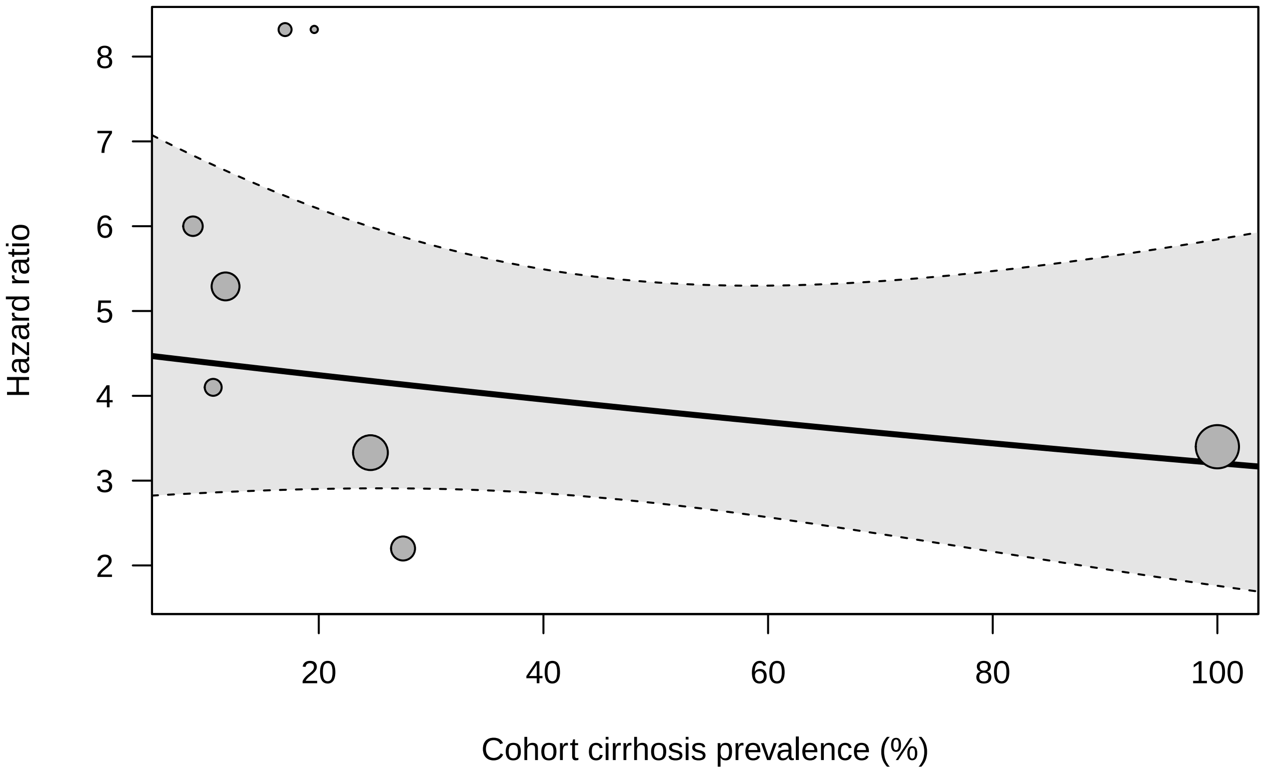
Hazard ratio
8
7
6
5
4
3
2
20
40
60
80
100
Cohort cirrhosis prevalence (%)

Supplementary Table S1. Non-viral M2BPGi–HCC studies identified at full-text screening and excluded from the primary longitudinal synthesis.

Study	Etiology	Design	N (HCC)	M2BPGi–HCC effect	Reason not eligible for primary pool
Kawanaka 2018 [38]	NAFLD	Retrospective; multivariable logistic regression with cumulative-incidence follow-up (>2 y)	331 (51)	OR 1.57 (95% CI 1.083–2.265) per COI; AUROC 0.806 (cut-off 1.255 COI)	Odds ratio from logistic regression (not a time-to-event hazard ratio); continuous per-COI estimate; non-viral etiology
Sasaki 2025 [39]	Non-viral chronic liver disease with type 2 diabetes	Cross-sectional retrospective (HCC vs non-HCC comparison)	330	M2BPGi ≥0.9 COI independently associated with HCC; in patients ≥65 y, OR 9.05 for M2BPGi ≥1.18 COI	Cross-sectional design; odds ratio; non-viral etiology

AUROC, area under the receiver operating characteristic curve; COI, cut-off index; HCC, hepatocellular carcinoma; NAFLD, non-alcoholic fatty liver disease; OR, odds ratio. Both studies were identified at full-text screening and excluded from the primary longitudinal hazard-ratio synthesis because they did not report a longitudinal hazard ratio with a categorical M2BPGi cut-off. A literature search updated to June 2026 identified no non-viral longitudinal cohort reporting a hazard ratio for incident HCC with M2BPGi.

Supplementary Table S2. Study-specific M2BPGi cut-off and cohort cirrhosis prevalence in the 15 primary (longitudinal, categorical, hazard-ratio) studies, with meta-regression results.

Study	Etiology	Cut-off (COI)	Cirrhosis (%)	HR (95% CI)
Ichikawa 2017	CHB	0.71	19.6	8.32 (1.03–67.0)
Shinkai 2018	CHB	1.215	NR	5.07 (1.69–15.19)
Murata 2020	CHB	1.5	NR	34.9 (4.3–284.9)
Chen 2024	CHB	1.5	100	3.40 (1.779–6.511)
Tseng 2020	CHB	1.73	NR	2.12 (1.17–3.86)
Takemura 2020	HCV	1.62	NR	7.78 (2.66–22.77)
Sato 2021	HCV	1.62	NR	12.565 (3.501–45.092)
Yasui 2018	HCV	1.75	8.8	6.0 (1.8–19.4)
Nakagawa 2020	HCV	1.8	27.5	2.20 (0.79–6.15)
Osawa 2020	HCV	1.85	11.7	5.29 (2.07–13.0)
Sasaki 2015	HCV	2.0	NR	5.71 (1.66–19.57)
Sato 2016	HCV	2.80	NR	15.21 (1.77–130.94)
Yamasaki 2014	HCV	4.0	17.0	8.318 (1.784–38.791)
Chang 2026	HCV	4.0	24.6	3.33 (1.54–7.23)
Tamaki 2015	HCV	4.2	10.6	4.1 (1.1–15.0)

Meta-regression — cut-off (COI), k = 15: ratio of hazard ratios per 1-COI increase 0.96 (95% CI 0.66–1.39), p = 0.80; R^2 = 0%.

Meta-regression — cohort cirrhosis prevalence, k = 8: ratio of hazard ratios per 10% increase 0.97 (95% CI 0.89–1.05), p = 0.36; R^2 = 0%.

CHB, chronic hepatitis B; CI, confidence interval; COI, cut-off index; HCV, chronic hepatitis C; HR, hazard ratio; NR, not reported. Cut-off values are the study-specific thresholds used to define elevated M2BPGi. Cirrhosis prevalence is the cohort-level proportion; no study reported hazard ratios stratified by individual cirrhosis status, so a true cirrhotic-versus-non-cirrhotic subgroup meta-analysis was not feasible. Neither moderator significantly modified the pooled hazard ratio.
