# Supplementary material for: Serum M2BPGi as a predictor of hepatocellular carcinoma development in chronic hepatitis B and C: a systematic review and meta-analysis
Source: Front Med (Lausanne). 2026 Jul 15;13:1897953. doi: 10.3389/fmed.2026.1897953 (PMC13416451; doi:10.3389/fmed.2026.1897953)
Supplement: Supplementary file 2 [file Data_Sheet_2.docx]

# PRISMA 2020 Checklist

Manuscript: Serum M2BPGi as a Predictor of Hepatocellular Carcinoma: A Systematic Review and Meta-Analysis

| **Section/Topic** | **Item No** | **Checklist item** | **Location in manuscript** |
| --- | --- | --- | --- |
| **TITLE** | | | |
| **Title** | **1** | Identify the report as a systematic review. | Title page |
| **ABSTRACT** | | | |
| **Abstract** | **2** | See the PRISMA 2020 for Abstracts checklist. | Abstract |
| **INTRODUCTION** | | | |
| **Rationale** | **3** | Describe the rationale for the review in the context of existing knowledge. | Introduction |
| **Objectives** | **4** | Provide an explicit statement of the objective(s) or question(s) the review addresses. | Introduction (last paragraph) |
| **METHODS** | | | |
| **Eligibility criteria** | **5** | Specify the inclusion and exclusion criteria for the review and how studies were grouped for the syntheses. | Methods — Eligibility Criteria |
| **Information sources** | **6** | Specify all databases, registers, websites, organisations, reference lists and other sources searched or consulted to identify studies. Specify the date when each source was last searched or consulted. | Methods — Search Strategy |
| **Search strategy** | **7** | Present the full search strategies for all databases, registers and websites, including any filters and limits used. | Methods — Search Strategy; Supplementary |
| **Selection process** | **8** | Specify the methods used to decide whether a study met the inclusion criteria of the review, including how many reviewers screened each record and each report retrieved for eligibility, whether they worked independently, and if applicable, details of automation tools used in the process. | Methods — Study Selection |
| **Data collection process** | **9** | Specify the methods used to collect data from reports, including how many reviewers collected data from each report, whether they worked independently, any processes for obtaining or confirming data from study investigators, and if applicable, details of automation tools used in the process. | Methods — Data Extraction |
| **Data items** | **10a** | List and define all outcomes for which data were sought. Specify whether all results that were compatible with each outcome domain in each study were sought (e.g., for all measures, time points, numerical results), and if not, the methods used to decide which results to collect. | Methods — Data Extraction |
|  | **10b** | List and define all other variables for which data were sought (e.g., participant and intervention characteristics, funding sources). Describe any assumptions made about any missing or unclear information. | Methods — Data Extraction |
| **Study risk of bias assessment** | **11** | Specify the methods used to assess risk of bias in the included studies, including details of the tool(s) used, how many reviewers assessed each study and whether they worked independently, and if applicable, details of automation tools used in the process. | Methods — Quality Assessment |
| **Effect measures** | **12** | Specify for each outcome the effect measure(s) (e.g., risk ratio, mean difference) used in the synthesis or presentation of results. | Methods — Statistical Analysis |
| **Synthesis methods** | **13a** | Describe the processes used to decide which studies were eligible for each synthesis (e.g., tabulating the study intervention characteristics and comparing against the planned groups for each synthesis). | Methods — Statistical Analysis |
|  | **13b** | Describe any methods required to prepare the data for presentation or synthesis, such as handling of missing summary statistics, or data conversions. | Methods — Statistical Analysis |
|  | **13c** | Describe any methods used to tabulate or visually display results of individual studies and syntheses. | Methods — Statistical Analysis; Figure Legends |
|  | **13d** | Describe any methods used to synthesize results and provide a rationale for the choice(s). If meta-analysis was performed, describe the model(s), method(s) to identify the presence and extent of statistical heterogeneity, and software package(s) used. | Methods — Statistical Analysis |
|  | **13e** | Describe any methods used to explore possible causes of heterogeneity among study results (e.g., subgroup analysis, meta-regression). | Methods — Subgroup and Sensitivity Analyses |
|  | **13f** | Describe any sensitivity analyses conducted to assess robustness of the synthesized results. | Methods — Subgroup and Sensitivity Analyses |
| **Reporting bias assessment** | **14** | Describe any methods used to assess risk of bias due to missing results in a synthesis (e.g., funnel plot, statistical tests for funnel plot asymmetry, Egger's test). | Methods — Publication Bias |
| **Certainty assessment** | **15** | Describe any methods used to assess certainty (or confidence) in the body of evidence for an outcome. | N/A |
| **RESULTS** | | | |
| **Study selection** | **16a** | Describe the results of the search and selection process, including results of any automation tools at each stage, preferably with a flow diagram. | Results — Literature Search and Study Selection; PRISMA flow diagram |
|  | **16b** | Cite studies that might appear to meet the inclusion criteria but which were excluded, and explain why they were excluded. | Supplementary / Screening records |
| **Study characteristics** | **17** | Cite each included study and present its characteristics. | Results — Study Characteristics; Table 1 |
| **Risk of bias in studies** | **18** | Present assessments of risk of bias for each included study. | Results — Quality Assessment; Table 2 |
| **Results of individual studies** | **19** | For all outcomes, present, for each study: (a) summary statistics for each group (where appropriate) and (b) an effect estimate and its precision (e.g., confidence/credible interval), ideally with a forest plot. | Figures 1–3; Supplementary Figures 1–2 |
| **Results of syntheses** | **20a** | For each synthesis, briefly summarise the characteristics and risk of bias among contributing studies. | Results — Primary Meta-Analysis |
|  | **20b** | Present results of all statistical syntheses conducted. If meta-analysis was done, present for each the summary estimate and its precision, the amount of statistical heterogeneity and the test for heterogeneity. | Results — Primary Meta-Analysis; Table 3 |
|  | **20c** | If comparing groups, describe the direction of the effect. | Results — Subgroup Analyses |
|  | **20d** | Report results of any investigation of possible causes of heterogeneity among study results. | Results — Subgroup Analyses |
|  | **20e** | Report results of any sensitivity analyses conducted to assess robustness of the synthesized results. | Results — Sensitivity Analyses; Supplementary Figure 5 |
| **Reporting biases** | **21** | Present assessments of risk of bias due to missing results (arising from reporting bias) for each synthesis assessed. | Results — Publication Bias; Supplementary Figures 3–4 |
| **Certainty of evidence** | **22** | Present assessments of certainty (or confidence) in the body of evidence for each outcome assessed. | N/A |
| **DISCUSSION** | | | |
| **Discussion** | **23a** | Provide a general interpretation of the results in the context of other evidence. | Discussion |
|  | **23b** | Discuss any limitations of the evidence included in the review. | Discussion (Limitations paragraph) |
|  | **23c** | Discuss any limitations of the review processes used. | Discussion (Limitations paragraph) |
|  | **23d** | Discuss implications of the results for practice, policy, and future research. | Discussion (last paragraph) |
| **OTHER INFORMATION** | | | |
| **Registration and protocol** | **24a** | Provide registration information for the review, including register name and registration number, or state that the review was not registered. | Methods — Study Registration; Title page |
|  | **24b** | Indicate where the review protocol can be accessed, or state that a protocol was not prepared. | PROSPERO registration |
|  | **24c** | Describe and explain any amendments to information provided at registration or in the protocol. | N/A |
| **Support** | **25** | Describe sources of financial or other support for the review, and the role of the funders or sponsors in the review. | Declarations — Funding |
| **Competing interests** | **26** | Declare any competing interests of review authors. | Declarations — Conflicts of interest |
| **Availability of data, code and other materials** | **27** | Report which of the following are publicly available and where they can be accessed: template data collection forms; data extracted from included studies; data used for all analyses; analytic code; any other materials used in the review. | Declarations — Data availability |

From: Page MJ, et al. The PRISMA 2020 statement: an updated guideline for reporting systematic reviews. BMJ 2021;372:n71.
